# Fine-Root Distribution and Soil Physicochemical Property Variations in Four Contrasting Urban Land-Use Types in South Korea

**DOI:** 10.3390/plants13020164

**Published:** 2024-01-07

**Authors:** Lan Thi Ngoc Tran, Ji Young An, Mark Bryan Carayugan, Jonathan O. Hernandez, SK Abidur Rahman, Woo Bin Youn, Julia Inacio Carvalho, Min Seon Jo, Si Ho Han, Hai-Hoa Nguyen, Byung Bae Park

**Affiliations:** 1 Department of Forest Resources, College of Agriculture and Life Science, Chungnam National University, Daejeon 34134, Republic of Korea; tranlan1218@gmail.com (L.T.N.T.); macarayugan@up.edu.ph (M.B.C.); abidurahman188@gmail.com (S.A.R.); dbsdnqls95@cnu.ac.kr (W.B.Y.); juliaincarvalho@gmail.com (J.I.C.); mean_sun@knps.or.kr (M.S.J.); bupleurumhan@gmail.com (S.H.H.); 2 Division of Environmental and Forest Science, College of Agriculture and Life Sciences, Gyeongsang National University, Jinju 52725, Republic of Korea; 3 Department of Forest Biological Sciences, College of Forestry and Natural Resources, University of Philippines, Laguna 4031, Philippines; johernandez2@up.edu.ph; 4 Gyeryongsan National Park Office, Korea National Park Service, Gongju 32626, Republic of Korea; 5 Kasuya Research Forest, Kyushu University, Sasaguri, Fukuoka 811-2415, Japan; 6 Faculty of Forest Resources & Environmental Management, Vietnam National University of Forestry, Hanoi 10000, Vietnam; hoanh@vnuf.edu.vn

**Keywords:** fine-root vertical distribution, soil properties, soil depth, urban land-use types

## Abstract

Urbanization and associated forest conversions have given rise to a continuum of native (forest fragments) and modified (artificial grasslands and perennial ecosystems) land-use types. However, little is known about how these shifts affect soil and fine-root compartments that are critical to a functioning carbon and nutrient circulation system. In this study, soil physicochemical properties, fine-root mass, and vertical distribution patterns were investigated in four representative urban land-use types: grassland (ZJ), perennial agroecosystem (MP), broadleaf deciduous forest patch (QA), and coniferous evergreen forest patch (PD). We quantified the fine-root mass in the upper 30 cm vertical profile (0–30 cm) and at every 5 cm depth across three diameter classes (<2 mm, 2–5 mm, and <5 mm). Soil physicochemical properties, except for phosphorus, nitrogen, ammonium nitrogen, and sodium cations, varied significantly across land-use types. The total root biomass (<5 mm) decreased in the order of QA (700.3 g m^−2^) > PD (487.2 g m^−2^) > ZJ (440.1 g m^−2^) > MP (98.3 g m^−2^). The fine-root mass of ZJ and MP was correlated with soil nutrients, which was attributed to intensive management operations, while the fine-root mass of QA and PD had a significant relationship with soil organic matter due to the high inputs from forest litter. Very fine roots (<2 mm) presented a distinct decremental pattern with depth for all land-use types, except for MP. Very fine roots populated the topmost 5 cm layer in ZJ, QA, and PD at 52.1%, 49.4%, and 39.4%, respectively. Maintaining a woody fine-root system benefits urban landscapes by promoting soil stabilization, improving ground infiltration rates, and increasing carbon sequestration capacity. Our findings underscore the importance of profiling fine-root mass when assessing urban expansion effects on terrestrial ecosystems.

## 1. Introduction

Rapid population growth, together with growing resource demand and economic pressure, has driven urban expansion at unprecedented rates [1]. With more areas becoming urbanized, cities are projected to support 60% of the global population by 2030, with an increase to 70–86% by 2050 [2,3]. The surge in demand for urban space has fragmented vast areas of forest and transformed vegetated landscapes into a heterogeneous continuum of native (plantations and grasslands), slightly modified (agriculture), and artificial (roads and bare sites) land uses [4]. As of 2019, 60–85% of the world’s forests had been exposed to destructive anthropogenic activities, bringing adverse effects to soil quality, mineral nutrient storage, and ecosystem functions [5,6,7]. Soil physicochemical traits, such as moisture, pH, organic matter content, and nutrient concentration, are likely affected by land-use intensification due to surface soil depletion and deep soil layer exposure [8]. Forest land-use shifts also reduce mineral nutrient reservoirs and allocation to belowground plant compartments, which are critical for a functioning carbon and nutrient circulation system [9,10]. Investigating the effects of forest land-use change on these belowground pools is, thus, fundamental to the understanding of how urban transformation disrupts ecosystem functions and dynamics.

Fine roots are thin, actively expanding underground organs designed for water and nutrient acquisition [11]. Fine roots form an integral component of the net primary productivity (NPP) as they reflect the amount of plant carbon gained from photosynthesis [12,13]. As a representation of the total photosynthates allocated below ground, fine-root mass provides insights into how plants partition their biomass for growth and survival. Fine roots’ contribution to NPP could range from 10 to 60% in forest ecosystems [11,14], 24–87% in grasslands [15,16], and 12% in perennial agroecosystems [17,18]. Distinguished by their high degree of plasticity, rapid turnover rates, and synergistic relations with soil biotic communities, fine roots also help stabilize soil conditions and improve physicochemical traits like bulk density and porosity [19,20]. The vertical distribution of fine roots determines the spatial partition of soil carbon and nutrients that are vital to plant growth and productivity [11,21]. Ground infiltration, which relates to urban surface runoff and flooding during the rainy season, is also determined by the arrangement of fine-root masses across soil layers [22]. Fine-root distribution patterns are usually surface-concentrated, multidirectional, and dependent on various factors, including water and nutrient availability, species rooting habits, proximity to infrastructure, and root barriers [11].

Changes in soil properties, floristic composition, and structure due to land-use shifts have impacted fine-root mass growth and distribution patterns at fine ecosystem scales [23,24]. In modified landscapes (e.g., artificial grasslands and perennial agroecosystems), changes are intensified by management interventions in the form of soil amendment, tillage, and weed and pest control [25,26,27]. Previous studies seeking to understand fine-root mass at different pre- and post-conversion states independently reported a 230–800 g m^−2^ range for broadleaf and coniferous forests [11,28], 510–950 g m^−2^ for grass formations [11,29], and 192–637 g m^−2^ for agroecosystems [30,31]. As land-use shifts became more prevalent, studies comparing fine-root mass change across disturbance intensities started emerging. Transforming woody forests into grasslands or croplands, for instance, was reported to lower total fine-root mass by at least half [9,32]. Forest trees, with their deep taproots and laterally ramifying fine-root system, could sustain 3–6 times as much root mass compared to fibrous, less stabilizing grass roots [9,33]. On the other hand, the conversion of natural forests to perennial agroecosystems could reduce fine-root biomass by at least 70%, possibly as an outcome of drastic modifications in stand structure and vegetation dynamics [9,31,34]. Along an increasing disturbance gradient, [34] noted a 67–137 g m^−2^ difference between fine-root biomass in natural forests (357 g m^−2^) and agroforestry plantations (220–290 g m^−2^), which was linked to altered canopy structure and resulting changes in basal area, DBH, and tree height.

Similar to how fine roots contribute to soil, soil physicochemical properties also regulate fine-root mass accumulation patterns [35]. Bulk density and texture reflect two vital conditions for fine-root growth: the former indicates mechanical impedance, while the latter signals soil moisture and nutrient retention [36,37]. Fine roots penetrate less compact soils, settling in finely textured spaces with less likelihood of leaching. Soil pH affects nutrient availability and organic matter accumulation in mineral soils [38]. Soils with a low pH are likely to be associated with higher fine-root mass and more rapid growth rates relative to those with a high pH [34]. Meanwhile, soil nutrients, along with moisture, directly determine the rates of fine-root emergence and colonization in mineral soils [39]. Fertile soils stimulate the production of metabolically active fine roots [39] that grow faster, absorb more nutrients, and ramify in wider soil volumes [9]. Fertile soils additionally hasten fine-root turnover, which regulates the layer of active fine-root mass during growing seasons [40]. However, much remains unknown as to how fine-root mass is affected by land-use-driven changes in soil properties and vegetation structure. Spatial fine-root patterns by depth are also poorly contextualized in highly heterogeneous urban environments.

In this study, we investigated the quantity and vertical distribution patterns of fine-root mass for four vegetated land-use types in a representative urban region in South Korea. The land-use types were classified according to the vegetation or growth form (i.e., trees, crops, and grass) dominant at each site. We hypothesized that the fine-root biomass and necromass vary across land-use types, influenced by soil conditions and vegetation structure. Moreover, we predicted that the distribution patterns of fine roots differ across land-use types due to soil property variations. Investigating fine-root mass distributions by urban land-use type, coupled with an exploration of the relationships of fine-root mass with soil properties, contributes to the understanding of belowground dynamics and the refining of management strategies for sustainable urban development.

## 2. Results

### 2.1. Variation in Soil Properties by Land-Use Type

The soil texture was sandy loam, except for ZJ, and the soil bulk density was higher in MP than in PD. The thickness of the organic layer was 6 cm in PD, 4 cm in QA, 2 cm in ZJ, and less than 1 cm in MP. Soil physicochemical properties, except for available phosphorus (AP), total nitrogen (TN), ammonium nitrogen (NH_4_^+^-N), and sodium cation (Na^+^; *p* = 0.106–0.297), varied significantly with urban land-use type (*p* < 0.01 for NO_3_^−^-N, BD, sand, and clay; *p* < 0.001 for remaining soil traits). QA and PD had a more acidic and organic matter-rich soil than ZJ and MP. Soil pH showed a strong negative correlation with organic matter (r = −0.95; *p* < 0.05). Electrical conductivity (EC) and carbon/nitrogen ratio (C/N ratio) at the upper 30 cm depth appeared to be highest in PD and QA and lowest in MP and ZJ. Nitrate nitrogen (NO_3_^−^-N) decreased across land-use types, following the order of ZJ > MP > PD > QA. ZJ and MP contained more exchangeable cations relative to PD and QA. Potassium ions (K^+^) were higher in ZJ and MP by 3–5 times at 0–10 cm depth and by 2–3 times at 10–30 cm depth. Calcium ions (Ca^2+^) varied between forest and intensively managed soils by 1.6–2.9 cmol_c_ kg^−1^ at 0–10 cm and 2.2–2.9 cmol_c_ kg^−1^ at 10–30 cm, whereas magnesium ions (Mg^2+^) varied by 0.5–1.2 cmol_c_ kg^−1^ at 0–10 cm and 0.5–1.1 cmol_c_ kg^−1^ at 10–30 cm. Meanwhile, QA soils presented the lowest amount of aluminum ions (Al^3+^) at 0.04 cmol_c_ kg^−1^.

### 2.2. Edaphic Drivers of Fine-Root Mass

**Figure 1:**
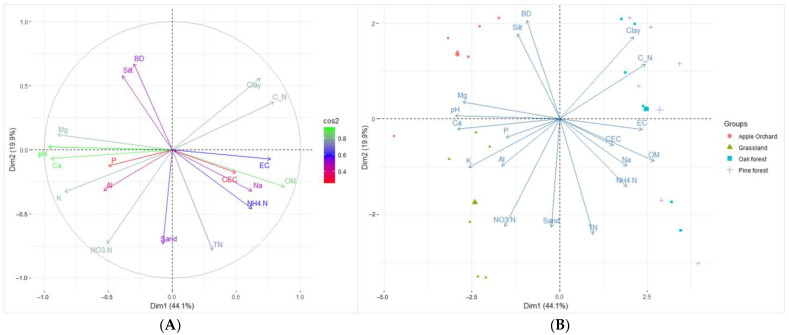
Principal component analysis of soil physicochemical variables (**A**) and fine-root mass observations (**B**) in artificial grassland (ZJ), apple orchard (MP), *Q. acutissima*-dominated forest (QA), and *P. densiflora*-dominated forest (PD) in Daejeon, Republic of Korea. SBD, soil bulk density; EC, electrical conductivity; OM, organic matter; P, available phosphorus; TN, total nitrogen; C/N, carbon-to-nitrogen ratio; NH_4_^+^-N, ammonium nitrogen; NO_3_^−^-N, nitrate nitrogen; CEC, cation-exchange capacity; K^+^, Na^+^, Ca^2+^, Mg^2+^, Al^3+^, exchangeable cations.

The relationship of fine-root mass with soil traits was projected onto a two-dimensional plot using PCA. The first two axes, partitioning one urban land-use type from another, captured 63.6% of the total fine-root mass variation (Figure 1). PC1 (eigenvalue = 7.93) had a strong positive loading for OM (0.87) and strong negative loadings for pH (−0.96), Ca^2^ (−0.94), and Mg^2+^ (−0.89). Bulk density (0.66), TN (−0.78), and NO_3_^−^-N (−0.72) loaded strongly onto PC2, which had a 3.59 eigenvalue. PC3 (eigenvalue = 2.45) explained an additional 14.03% of the overall variation, relating positively to silt (0.67) and negatively to sand (−0.62). Fine-root mass from intensively managed landscapes populated the left side of the biplot and correlated with soil fertility. In contrast, observations from the forest sites settled on the right side, relating more substantially to OM. This partition reflects the fertile soil conditions in ZJ and MP, as well as the OM abundance in QA and PD.

### 2.3. Variations in Standing Fine-Root Biomass and Necromass across Four Land-Use Types

The mean biomass of <5 mm roots ranged from 98.3 to 700.3 g m^−2^, with significant differences across depth and urban land-use types (*p* < 0.001; Table 1). The fine-root biomass of ZJ was 341.8 g m^−2^ higher than that of MP but 388.9–602.1 g m^−2^ lower than that of forest landscapes in the upper 30 cm depth. Variation by urban land-use type was prominent among very fine roots (*p* < 0.001) while indiscernible among small ones (*p* = 0.054). Fine-root necromass varied significantly by depth and urban land-use types for all diameter classes (*p* < 0.001). Fine roots with a diameter of <5 mm decreased in the order of QA (64.5 g m^−2^) > ZJ (88.5 g m^−2^) > PD (34.33 g m^−2^) > MP (1.06 g m^−2^) at 0–30 cm soil depth. Small roots contributed 92% to the total fine-root necromass in ZJ, while very fine roots accounted for 70.5% in QA. Biomass components occupied a larger portion of the total fine-root biomass than necromass, with differences of 351.6 g m^−2^ for ZJ, 97.2 g m^−2^ for MP, 452.9 g m^−2^ for PD, and 635.8 g m^−2^ for QA.

### 2.4. Vertical Distribution of Fine-Root Biomass and Necromass in Four Land-Use Types

**Figure 2:**
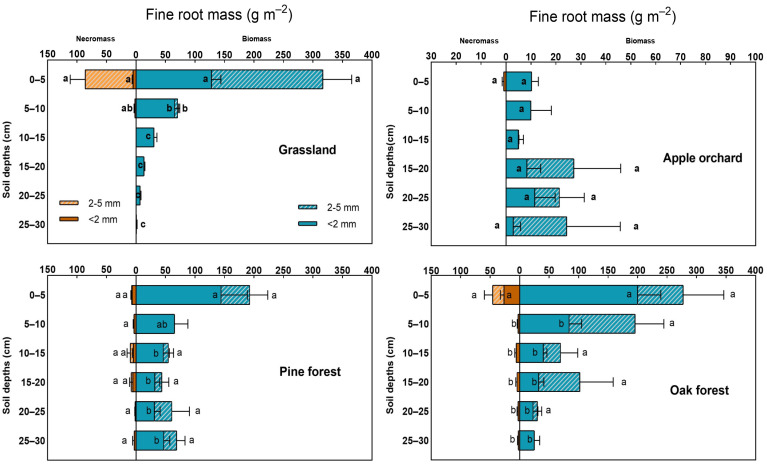
Vertical distribution of fine-root biomass and fine-root necromass (g m^−2^) across different root diameters at the four sites in Daejeon, Republic of Korea. Bars represent the mean standard error per depth interval for each land-use type (*n* = 5). Different lowercase letters along soil depth indicate statistically significant differences (*p* < 0.05).

The depth-wise pattern of fine-root biomass and necromass was specific to each site. Very-fine-root biomass showed a distinct decremental pattern with depth in all land-use types, except for MP, whose distribution was uniform across layers (Figure 2). In ZJ, 52.1% of very fine living roots crowded the first 5 cm of the mineral soil, decreasing abruptly to 26.6% at 5–10 cm, 12.4% at 10–15 cm, and 0.3–5.6% at 15–30 cm. In forest landscapes, the 0–5 cm layer hosted 49.4% of very fine living roots in QA and 39.4% in PD. Very-fine-root biomass decreased significantly past 5 cm, stabilizing at a 22.2–84.0 g m^−2^ range in QA and 31.2–65.2 g m^−2^ in PD. For small roots, ZJ showed a descending pattern across depth, which was otherwise homogenous in QA and PD. Small roots occupied the upper 5 cm of the mineral soil at 97.3%, leaving only 2.7% for the 5–10 cm layer. Among the land-use types, ZJ contained the largest amount of shallow fine roots (0–5 cm depth; 316.8 g m^−2^), followed by QA (277.3 g m^−2^), PD (193.1 g m^−2^), and then MP (10.3 g m^−2^). Fine roots of 5 cm to 30 cm, on the other hand, were abundant in QA (422.9 g m^−2^), intermediate in PD (294.1 g m^−2^) and ZJ (123.2 g m^−2^), and very little in MP (88.0 g m^−2^). The fine-root necromass of ZJ and QA decreased significantly with depth, while those of MP and PD appeared homogeneous across layers. Vertical fine-root necromass followed the same order as fine-root biomass when compared across land-use types, i.e., ZJ > QA > PD > MP at 0–5 cm and QA > PD > ZJ > MP at 5–30 cm. In ZJ, 97.6% of the fine-root necromass was concentrated at 0–5 cm, while the remaining 2.4% was at 5–10 cm. The fine-root necromass in QA was distributed as follows: 70.8% at 0–5 cm, 4.5% at 5–10 cm, 9.6% at 10–15 cm, 7.2% at 15–20 cm, 4.3% at 20–25 cm, and 3.6% at 25–30 cm. The decremental trend of fine-root necromass in QA and ZJ was prominent both among very fine roots and small roots for the former, but only among very fine roots for the latter.

## 3. Discussion

### 3.1. Variation in Soil Physical and Chemical Properties across Land-Use Types

Soil pH, organic matter levels, calcium, and potassium explained much of the soil property variations between forested (QA and PD) and intensively managed urban landscapes (ZJ and MP; Table 1). Soil pH reflects plant community interaction with the belowground environment and assumes an important role in organic matter accumulation, nutrient availability, and soil quality and health [41]. In this study, broadleaf and coniferous forest soils possessed a higher level of acidity than grass and perennial agroecosystem soils. The supply of essential nutrients for fine-root production and growth may have been limited by soil pH conditions, as is the case for apple orchards [42,43], grassland [42], and pine and oak forests [44].

As expected, PD and QA soils contained more organic matter compared to the soils of ZJ and MP. Forest trees contribute significantly to the inflow of organic compounds through litterfall production [45], deposition to the forest floor, and decomposition [46]. Intensively managed landscapes suffer from low organic matter due to the paucity (for MP) or absence (for ZJ) of dead and decomposing materials from trees. Vegetation structure affects the rate of organic matter accumulation in mineral soils. Differences in soil temperature, aeration, and mycorrhizal associations in the rhizosphere also widen the extent of variation across land-use types. For instance, a high basal area and stand density, similar to QA and PD, could reduce temperature and oxygen levels to the point of hastening organic matter formation [47,48]. Litter decomposition, a process partly determined by morpho-chemical properties, differs by land-use type. These differences in litter decomposition rate affect the release of organic compounds into mineral soils [49].

Edaphic factors like pH and texture regulate both the availability and abundance of calcium in intensively managed landscapes. In this study, calcium concentrations were higher in ZJ and MP than in QA and PD, which was attributed to area management interventions, high Ca^2+^ demand, and adaptive capacities (Figure 1 and Table 1). Grasslands generally host diverse species thriving in calcium-abundant soils, supported by research showing their favorable soil conditions (e.g., pH and texture) for essential nutrient release, including calcium [42]. Apple trees utilize a significant amount of calcium during fruit production, which necessitates annual soil amendment [50]. Intensive management practices, usually in the form of co-compost application, pruning, and vegetation control, also contribute to the high macronutrient concentration in MP soils. Achieving optimal root growth in perennial agroecosystems, therefore, entails proper soil management and tree maintenance. On the other hand, acidic soil conditions and natural rooting habits could explain the low calcium levels observed in PD and QA. Despite having deep taproots and an active lateral fine-root system [51,52], *P. densiflora* and *Q. acutissima* absorb little calcium since most soluble Ca^2+^ leaches out of the soil under low pH environments. Pine and oak trees may have adapted to such limitations by harnessing alternative nutrient sources for root growth and development.

Potassium facilitates root growth by promoting cell expansion and phloem transport [53,54]. For instance, low potassium solubility impairs the nutrient-absorptive function of a *Pinus sylvestris* forest [53]. In the present study, the demand for potassium varied across dominant species located in four land-use types, with grasslands and agroecosystems needing higher amounts for growth than unmanaged forests. The high potassium level in ZJ and MP likely stems from the frequent application of co-compost and potassium salt-rich pesticides, which hastens K^+^ accumulation in soil and plant tissues. In contrast, forest landscapes lack an external source and have slow nutrient turnover rates [55], thus showing a lower K^+^ concentration in the soil (Table 1).

### 3.2. Variation in Standing Root Mass and Fine-Root Vertical Distribution across Land-Use Types

Our results coincide with those of previous investigations on fine-root mass distribution in forest trees, grasses, and perennial fruit crops. In a previous study examining a limestone forest in Southwest China, the total fine-root mass ranged from 187 to 303.1 g m^−2^ and consisted of 137–216.2 g m^−2^ biomass and 47.3–86.9 g m^−2^ necromass [56]. However, our findings were lower than the fine-root biomass (800 g m^−2^) and necromass (90 g m^−2^) reported in the upper 30 cm profile of a *Quercus serrata* Roxb. forest in Northern Japan [28]. Ref. [11] synthesized live fine-root mass data for global temperate forest formations and reported a 440 g m^−2^ estimate in temperate deciduous forests, 500 g m^−2^ in temperate coniferous forests, and 950 g m^−2^ in temperate grasslands. As per recent studies, biomass estimates could range from 354 to 658.5 g m^−2^ (0–10 cm) in temperate deciduous forests, while biomass and necromass values could reach 523 g m^−2^ and 186 g m^−2^ in temperate coniferous forests, respectively [57].

Root distribution patterns in urban areas varied by land-use type, which was consistent with our hypothesis. An analysis of the vertical fine-root profile revealed that grass roots thrive in shallow depths and grow rarely in deeper soil layers. Their preference for shallow depth was exemplified by [58], who reported 67.5% of total live biomass in the first 10 cm of mineral soil. This indicates that fine-root growth habits and soil properties determine the arrangement of fine roots along a vertical space. Grass species like *Z. japonica* develop rhizomatic roots, a specialized organ consisting of nodal series and connecting shoots that expand and continuously form new connections [59]. This root system is responsible for horizontal root growth as well as fine-root proliferation within superficial layers [60]. Besides rooting habits, grassland management operations like mowing regulate root distribution patterns through their influence on soil physicochemical conditions. Ref. [16] found that mowing substantially reduced the rooting depth of warm-season grasses in northern China. Frequent mowing operations, i.e., four times a year, possibly generated enough nitrogen-rich residues to change the soil chemical properties and, consequently, affect the fine-root distribution in ZJ. The inconspicuous vertical pattern in MP contradicts studies that reported high fine-root biomass activity at 20–40 cm and even at lower depths for old *M. pumila* trees [42,61]. The absence of a vertical trend in apple orchards may be due to the mixing effect of soil plows. Meanwhile, the stand structure, canopy traits, and fine-root morphological characteristics likely explain the fine-root mass variations in PD and QA. The basal area and tree density were higher in QA (43.7 m^2^ ha^−1^ and 900 tree ha^−1^) than PD (39.4 m^2^ ha^−1^ and 800 tree ha^−1^, Table 2), indicating that QA soils receive more organic matter from litter (Table 1 and Figure 1) and could stimulate fine-root growth better than PD soils. Further, dense forests have a large belowground absorption capacity owing to their high standing fine-root biomass.

All sites, except for MP, showed a decreasing fine-root mass response with depth, aligning well with reports on different vegetation types (Figure 2). Many studies have shown a retreating fine-root pattern across depth [28,62,63], which carries important implications for nutrient uptake. Among landscapes dominated by trees, the fine-root biomass of QA decreasedsharply with depth, partly due to the high subcanopy diversity. The authors of [63] investigated standing fine-root mass and production along a diversity gradient in China and found that species-rich communities had a more distinct vertical fine-root response than their less diverse counterparts. Moreover, the shallow rooting habits of understory shrubs may supplement the already high fine-root mass quantity in the upper soil layers. The authors of [64] found that shrubs contributed more absorptive roots to the topsoil than woody vegetation. Although we did not classify fine roots up to the species level, the vertical fine-root pattern of understory plants revealed greater substrate exploitation in the upper and most nutrient-abundant mineral soil layer. In species-complex landscapes, layers where canopy and subcanopy plants populate their fine roots can vary strategically; such a mechanism can be beneficial as it avoids belowground competition and broadens fine-root mass quantities across depth [65].

### 3.3. Implications for Sustainable Urban Planning

This study underscores the value of fine-root mass measurement and vertical profiling when evaluating urban ecosystem health. High fine-root densities, such as those noted for grasslands and forest landscapes, may signal a more floristically complex and stable ecosystem [66]. These findings could guide urban planners in selecting appropriate land use for a given area and refining the conservation aspect of urban green space management. Based on our findings, it is recommended to prioritize expanding forest patches and grasslands in urban ecological areas and peri-urban zones. Allocating space for belowground growth and root diversification offers multiple ecosystem benefits, ranging from increased soil infiltration capacity to reduced heat-island effects and minimized stormwater runoff, among many others [22,33]. Further, determining how fine roots are distributed along vertical spaces can help urban systems cope with the consequences of sustained stoichiometric imbalances caused by destructive anthropogenic activities [67].

Forests are robust systems for carbon absorption, with organic matter forming a large fraction of the terrestrial carbon pools. Landscapes intended for carbon capture are relevant to mitigate climate change and improve air quality in urban zones [33]. Understanding the fine-root distribution in different vegetated formations facilitates the identification and selection of high-carbon storage areas, thus facilitating the maintenance or, better yet, the expansion of key sequestration zones [68]. Supported by our results, we suggest integrating vegetated systems into urban regions to enhance climate mitigation capacities and to usher in sustainable urban development.

## 4. Materials and Methods

### 4.1. Study Site

**Figure 3:**
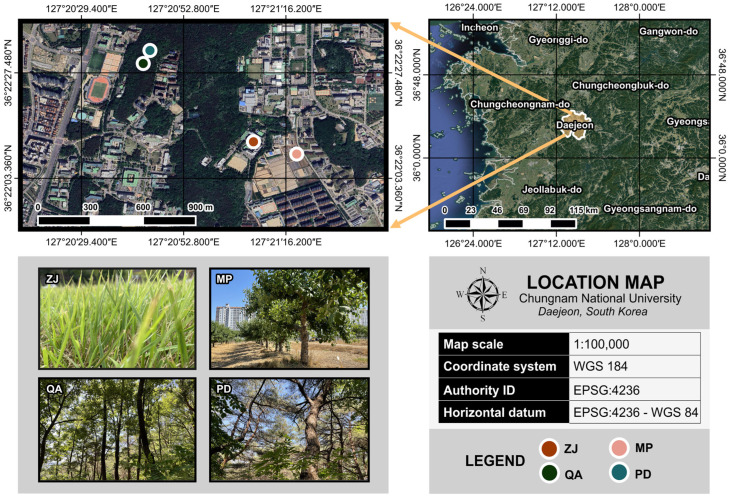
Geographical location of the four study sites in Daejeon, Republic of Korea. ZJ, MP, QA, and PD represent artificial grassland, apple orchard, *Quercus acutissima*-dominated forest, and *Pinus densiflora*-dominated forest, respectively. All geospatial vectors and coordinates are illustrated via EPSG 4236-WGS 84 horizontal datum.

This study was conducted in Daejeon, the fifth largest city and a major hotspot for technology, transportation, research, and development in the central region of South Korea. We situated our research sites at Chungnam National University (80–110 masl), a 160-ha area located near the city center, as it lies close to the city’s major industrial complexes and, at the same time, harbors a range of well-characterized urban ecological formations, from grasslands and perennial agroecosystems to broadleaf deciduous and coniferous evergreen forest patches (Figure 3). Confining our research sites to one suitable location ensures that each urban land-use type shares a fairly homogenous topography and climate, which, by themselves, could be a source of unwanted variation. The research sites host a hot humid continental climate (Dfa in the Köppen–Geiger system) distinguished by approximately 27 °C in warm summers from June to August and −0.9 °C in cold winters from December to February, based on 2020–2021 climate data. The annual normal temperature averaged 13.1 °C from 2010 to 2020, and the annual precipitation totaled 1351.2 mm. The maximum rainfall occurred in July at 496 mm, while the minimum took place in January at 1.2 mm. The dominant soil type is brown forest soil equivalent to Alfisol in the USDA taxonomy.

We measured the fine-root mass and soil physicochemical traits in four representative urban landscapes: grassland (36°22′11.7″ N, 127°21′08.8″ E; *hereafter*, ZJ), perennial agroecosystem (36°22′08.888″ N, 127°21′18.7″ E; *hereafter*, MP), coniferous evergreen forest patch (36°22′33.6324″ N, 127°20′43.2744″ E; *hereafter*, PD), and broadleaf deciduous forest patch (36°22′18.4800″ N, 127°20′43.4292″ E; *hereafter*, QA) (Table 3). ZJ is a non-grazing, perennial grassland comprising finely bladed *Zoysia japonica* Steud. leaves aboveground and a fibrous, dense, and thick network of shallow roots belowground. Grass measures roughly 20 cm in height and 140 g m^−2^ in aboveground biomass. ZJ is maintained through periodic mowing and herbicide spraying. Mowing operations take place four times per year, i.e., once in May, July, August, and October, with the mowing height fixed at 2–3 cm. Grass residues from mowing are left in the area until fully decomposed. ZJ receives a selective, systemic herbicide once a year in April. MP is an intensively managed, perennial agroecosystem that specializes in *Malus pumila* Mill. fruit production. Its belowground system consists of extensive, fibrous, and laterally ramifying fine-root systems. Major site maintenance operations in the area include weeding, irrigation, and pesticide and compost applications. *M. pumila* trees receive water from drip irrigation during intense dry spells in the summer. Fruits grown in September–October are sprayed with pesticides to avoid damage from pests. At least 25 kg of sawdust × manure co-compost, made up of 2% nitrogen, 0.5–1% phosphorus, and 2% potassium, is applied to the surface of each tree every year in December. PD is a coniferous evergreen stand dominated by *Pinus densiflora*, a species with fine feeder roots and a deep taproot system. Its subcanopy stratum supports a host of plant species, including *Cornus officinalis*, *Robinia pseudoacacia*, *Prunus sargentii*, *Quercus serrata*, *Alnus hirsuta*, and *Eunymous sachalensis*. Finally, QA is a broadleaf deciduous stand represented by *Quercus acutissima* in the canopy. This dominant canopy species is known for its thick vertical taproots designed for ground anchorage as well as its dense, actively expanding lateral root system that serves as an apparatus for resource uptake. QA has a subcanopy layer consisting of *Castanea pumila*, *Quercus crispula*, *Magnolia kobus*, *Prunus sargentii*, *Robinia pseudoacacia*, *Juniperus rigida*, and *Magnolia obovata.* We sampled a 400 m^−2^ area at each site for a comprehensive vegetation inventory, measuring, wherever possible, representative species at each stratum, height, DBH, basal area, and tree density.

### 4.2. Soil Sampling

We obtained six soil samples from two depth classes (0–10 cm and 10–30 cm) at three random locations per site using a 100 cm^3^ stainless cylinder. Soil physical properties were measured using the following approaches: the hydrometer method at 30 °C for texture and the cylinder method for bulk density. Using a soil pH and electrical conductivity (EC) meter, soil pH and EC were measured by immersing the electrodes 1–2 cm into a 1:5 (*w*/*v*) soil-distilled water suspension. Soil organic matter (OM) was measured based on wet combustion via the Walkley–Black method, total nitrogen (TN) was measured based on 1 g of soil using a PrimacsSNC-100 TOC/TN analyzer (Skalar Analytical B.V, Breda, TheNetherlands), and available phosphorus was measured via the Bray No. 1 test. Ammonium nitrogen (NH_4_^+^-N) was determined based on Indophenol blue colorimetry, while nitrate nitrogen (NO_3_^−^-N) was measured using ion chromatography (Thermo Scientific Dionex ICS-5000 Ion Chromatography System, Dionex, Sunnyvale, CA, USA). Cation-exchange capacity (CEC) was determined via the Brown method upon extraction with 1N of HN_4_OAc and CH_3_COOH solution. Exchangeable cations (e.g., K^+^, Ca^2+^, Mg^2+^, Al^3+^, and Na^+^) were measured in 1N NH_4_OAc extract by means of inductively coupled plasma optical emission spectrometry (iCAP 7400 ICP-OES Analyzer, Qtegra, Thermo Fisher Scientific, Cleveland, OH, USA). All chemical extraction procedures were in line with the protocol of the National Institute of Agricultural Science and Technology (2000).

### 4.3. Fine-Root Mass Inventory

Before data collection in 2022, a 100 m reference line was established at a random location in each site. The reference line was marked every 20 m to facilitate the identification of collection points and the systematic retrieval of soil samples. One soil sample was collected at each point, totaling five per reference line, from the forest floor down to a 30 cm depth using a stainless-steel corer with a 5.3 cm diameter. Each sample was stratified into six depth classes of 5 cm intervals, i.e., 0–5 cm, 5–10 cm, 10–15 cm, 15–20 cm, 20–25 cm, and 25–30 cm, and then stored in resealable plastic bags at −5 °C until processing. In the laboratory, fine roots were extracted manually from the soil, washed under running water over a 200 µm test sieve, and then dichotomized into either biomass (living) or necromass (dead) based on their color, texture, and shape. Fine roots were considered living if they were elastic and flexible and the exterior was pale to light brown. On the other hand, fragile samples with traces of decay as well as a brown to black exterior were treated as dead roots. Living and dead roots were sorted further into two diameter classes: <2 mm and 2–5 mm. All root samples were oven-dried at 65 °C for 72 h and then weighed using a standard laboratory balance. In this study, we reported both cumulative (0–30 cm) and 5 cm depth-wise fine-root mass for roots of <2 mm (very fine roots), 2–5 mm (small roots), and <5 mm (total roots) in g m^−2^.

### 4.4. Statistical Analysis

We used one-way analysis of variance (ANOVA) to assess variations in soil physical and chemical traits across land-use types. The same approach was used to identify urban land-use effects on fine-root biomass and necromass for each diameter class. For this analysis, observations of biomass and necromass of roots of <2 mm (very fine roots), 2–5 mm (small roots), and <5 mm (total roots) diameter were independently assigned as response variables, with land use as the explanatory variable. A two-way ANOVA was performed to evaluate the response of very fine and small roots across depths and land-use types. The individual effect of each variable was tested using a one-way ANOVA in cases of implicated interaction. All significantly different means were compared for urban land-use type, depth, or their interaction using Tukey’s honestly significant difference test. The data were subject to logarithmic transformation to satisfy the assumptions of normality and homoscedasticity. All statistical analyses, except for the principal component analysis (PCA), were performed using SPSS Statistics for Windows ver. 26.0 (SPSS Inc., Chicago, IL, USA) at a minimum 0.05 significance level. To summarize the interrelation of fine roots and soil physicochemical traits for the four urban land-use types, we performed a PCA using R for Windows ver. 4.2.2. Observations of soil physical traits (sand, silt, clay, and bulk density), soil chemical traits (pH, EC, OM, TN, AP, C/N ratio, NH_4_^+^-N, NO_3_^−^-N, CEC, K^+^, Ca^2+^, Mg^2+^, Al^3+^, and Na^+^), and fine-root mass were used to fine-tune the resulting plot.

## 5. Conclusions

Changes in soil conditions and vegetation structure due to forest conversions altered the fine-root mass and vertical distribution patterns at the landscape level. Soil fertility and organic matter differentiate the fine-root biomass and necromass quantities between forested (i.e., broadleaf deciduous and coniferous forest patches) and intensively managed landscapes (i.e., grassland and apple orchard). While the role of soil factors in fine-root traits warrants further investigation, our findings shed light on the influence of land-use shifts on fine-root quantity and distribution, which is relevant to the understanding of how ecosystems respond to global environmental changes. Further, this study helps pinpoint the factors (soil physicochemical properties, soil depth, etc.) regulating the spatial fine-root distribution of different plant forms, providing implications for sustainable management of vegetated landscapes under highly heterogeneous and continuously changing urban environments.

## Figures and Tables

**Table 1:** Soil physical and chemical properties measured in four land-use types in Daejeon, Republic of Korea. Mean standard errors are expressed in parenthesis (*n* = 3). Different lowercase letters within a column denote statistically significant differences among land-use types (*p* < 0.05).

	Physical Properties													
Sites	Sand			Silt			Clay			Soil Texture		SBD		
**ZJ**	a			b			c					ab		
0–10 cm	87 (0)			10 (0)			3 (0.01)			sand		1.27 (0.07)		
10–30 cm	85 (0.02)			11 (0.02)			4 (0)			loamy sand		1.41 (0.07)		
**MP**	c			a			b					a		
0–10 cm	65 (0.01)			27 (0.01)			8 (0.01)			sandy loam		1.52 (0.09)		
10–30 cm	67 (0.01)			25 (0.01)			8 (0)			sandy loam		1.58 (0.07)		
**QA**	b			b			b					ab		
0–10 cm	78 (0.01)			13 (0.01)			9 (0.01)			sandy loam		1.43 (0.07)		
10–30 cm	73 (0.03)			16 (0.01)			11 (0.02)			sandy loam		1.45 (0.09)		
**PD**	ab			b			a					b		
0–10 cm	73 (0.05)			13 (0.02)			13 (0.02)			sandy loam		1.23 (0.06)		
10–30 cm	69 (0.04)			15 (0.03)			16 (0.01)			sandy loam		1.37 (0.09)		
	**Chemical Properties**													
										**Exchangeable Cations**				
	**pH**	**EC**	**OM**	**AP**	**TN**	**C/N**	**NH_4_^+^-N**	**NO_3_^−^-N**	**CEC**	**K^+^**	**Na^+^**	**Ca^2+^**	**Mg^2+^**	**Al^3+^**
**ZJ**	a	c	c			b		a	b	a		a	b	a
0–10 cm	6.23	0.07	2.66	121.2	0.1	12.24	2.06	16.86	3.74	0.36	0.12	2.28	0.57	0.1
	(0.07)	(0.01)	(0.43)	(51.2)	(0.01)	(0.88)	(0.13)	(3.04)	(0.18)	(0.01)	(0.02)	(0.22)	(0.04)	(0.00)
10–30 cm	6.23	0.05	0.91	39.4	0.02	14.16	1.73	10.23	4.41	0.23	0.09	3.19	0.56	0.1
	(0.06)	(0.00)	(0.14)	(25.3)	(0.00)	(1.49)	(0.34)	(2.25)	(0.46)	(0.07)	(0.03)	(0.09)	(0.12)	(0.00)
**MP**	a	bc	c			b		b	b	a		a	a	a
0–10 cm	6.9	0.23	0.23	296.5	0.06	10.63	2.25	7.6	4.92	0.31	0.08	3.31	1.25	0.1
	(0.48)	(0.07)	(0.07)	(240.6)	(0.02)	(0.92)	(0.20)	(2.38)	(0.99)	(0.10)	(0.01)	(0.68)	(0.25)	(0.00)
10–30 cm	6.85	0.13	0.13	62.6	0.04	10.58	2.29	4.3	4.4	0.27	0.12	2.86	1.1	0.1
	(0.46)	(0.02)	(0.02)	(29.7)	(0.01)	(0.94)	(0.34)	(0.34)	(0.20)	(0.05)	(0.03)	(0.06)	(0.06)	(0.00)
**QA**	b	ab	b			a		b	a	b		b	c	b
0–10 cm	4.4	0.34	4.16	23.1	0.07	38.65	3.54	3.94	7.88	0.08	0.33	0.63	0.05	0.04
	(0.16)	(0.08)	(0.71)	(0.8)	(0.02)	(5.96)	(1.10)	(1.22)	(0.53)	(0.00)	(0.04)	(0.05)	(0.00)	(0.01)
10–30 cm	4.55	0.28	4.22	12.4	0.06	49.22	3.1	3.45	7.2	0.1	0.41	0.62	0.05	0.03
	(0.07)	(0.06)	(0.89)	(1.0)	(0.03)	(10.41)	(1.34)	(1.49)	(1.13)	(0.02)	(0.17)	(0.07)	(0.00)	(0.01)
**PD**	b	a	a			a		b	ab	b		c	c	a
0–10 cm	4.18	0.46	6.86	27.2	0.11	39.45	5.29	5.92	5.82	0.09	0.21	0.44	0.05	0.12
	(0.06)	(0.04)	(0.73)	(1.0)	(0.02)	(3.72)	(0.89)	(0.99)	(1.56)	(0.01)	(0.12)	(0.09)	(0.00)	(0.02)
10–30 cm	4.36	0.33	5.32	24.0	0.05	70.05	2.29	2.39	4.56	0.09	0.14	0.32	0.06	0.05
	(0.05)	(0.04)	(0.79)	(8.5)	(0.01)	(2.55)	(0.42)	(0.48)	(1.68)	(0.02)	(0.07)	(0.12)	(0.01)	(0.01)

SBD, soil bulk density (g cm^−3^); EC, electrical conductivity (dS m^−1^); OM, organic matter (%); AP, available phosphorus (mg kg^−1^); TN, total nitrogen (%); C/N, carbon-to-nitrogen ratio; NH_4_^+^-N, ammonium nitrogen (mg kg^−1^); NO_3_^−^-N, nitrate nitrogen (mg kg^−1^); CEC, cation-exchange capacity (cmolc kg^−1^); K^+^, Na^+^, Ca^2+^, M^2+^, Al^3+^, exchangeable cations (cmolc kg^−1^). ZJ, artificial grassland; MP, apple orchard; QA; *Quercus acutissima*-dominated forest; PD, *Pinus densiflora*-dominated forest.

**Table 2:** Fine-root biomass and necromass (g m^−2^) in four contrasting land-use types in Daejeon, Republic of Korea, and the results of two-way ANOVA showing the influence of land-use type, soil depth, and their interaction on fine-root biomass and necromass. The values in the parenthesis are the mean standard errors (*n* = 5). Different lowercase letters within a column denote statistically significant differences across land-use types (*p* < 0.05).

Site	Biomass (g m^−2^)				Necromass (g m^−2^)		
	<2 mm		2–5 mm	<5 mm	<2 mm	2–5 mm	<5 mm
**Fine root mass**							
ZJ	246.24 (17.23) ^a^		193.85 (50.69) ^a^	440.09 (65.28) ^ab^	7.18 (1.90) ^bc^	81.31 (25.17) ^a^	88.49 (25.29) ^a^
MP	48.25 (21.28) ^b^		50.05 (32.2) ^a^	98.30 (47.48) ^b^	1.06 (0.57) ^c^	0 ^b^	1.06 (0.57) ^b^
QA	404.87 (56.82) ^a^		295.43 (117.81) ^a^	700.30 (153.69) ^a^	45.46 (9.51) ^a^	19.04 (14.22) ^b^	64.49 (8.59) ^a^
PD	366.77 (55.10) ^a^		120.44 (30.91) ^a^	487.21 (57.35) ^a^	25.62 (7.17) ^ab^	8.71 (6.01) ^b^	34.33 (12.62) ^ab^
**ANOVA Summary**							
Variation source	*df*	<2 mm	2–5 mm	<5 mm	<2 mm	2–5 mm	<5 mm
Site	3	<0.0001	0.054	<0.001	<0.0001	0.001	0.001
Soil depth	5	<0.0001	0.002	<0.001	<0.0001	<0.001	<0.001
Site x soil depth	15	<0.0001	0.003	<0.001	<0.0001	<0.001	<0.001

ZJ, artificial grassland; MP, apple orchard; QA, *Quercus acutissima*-dominated forest; PD, *Pinus densiflora*-dominated forest.

**Table 3:** Structural description of four sites chosen for fine root mass sampling in Daejeon, Republic of Korea.

Site	ZJ	MP	QA	PD
Tree density (tree ha^−1^)	NA	700	900	800
Mean height (m)		3.7	13.8	12.5
Mean DBH (cm)		15.5	30.3	24.2
Basal area (m^2^ ha^−1^)		13.4	43.7	39.4
Dominant species	*Zoysia japonica*	*Malus pumila*	*Quercus acutissima*	*Pinus densiflora*

ZJ, artificial grassland; MP, apple orchard; QA; *Quercus acutissima*-dominated forest; *Pinus densiflora*-dominated forest. Structural parameter estimates are based on a 400 m^2^ plot inventory of standing canopy trees for PD and QA, row-wise inventory of standing apple trees for MP, and quadrat sampling for ZJ. NA indicates data are not available.

## Data Availability

The data presented in this study are available within the article.
